# Functional microarray analysis suggests repressed cell-cell signaling and cell survival-related modules inhibit progression of head and neck squamous cell carcinoma

**DOI:** 10.1186/1755-8794-4-33

**Published:** 2011-04-13

**Authors:** Anna EL Coló, Ana CQ Simoes, André L Carvalho, Camila M Melo, Lucas Fahham, Luiz P Kowalski, Fernando A Soares, Eduardo J Neves, Luiz FL Reis, Alex F Carvalho

**Affiliations:** 1 Hospital AC Camargo, Rua Taguá, 440, São Paulo, SP, 01508-010, Brazil; 2 Hospital Israelita Albert Einstein, Avenida Albert Einstein, 627, São Paulo, SP, 05652-900, Brazil; 3 Universidade Federal do ABC, Rua Santa Adélia, 166, Santo André, SP, 09210-170, Brazil; 4 Instituto de Matemática e Estatística da Universidade de São Paulo, Rua do Matão, 1010, São Paulo, SP, 05508-090, Brazil; 5 Hospital Sírio-Libanês, Rua Adma Jafet, 91, São Paulo, SP, 01308-050, Brazil

## Abstract

**Background:**

Cancer shows a great diversity in its clinical behavior which cannot be easily predicted using the currently available clinical or pathological markers. The identification of pathways associated with lymph node metastasis (N+) and recurrent head and neck squamous cell carcinoma (HNSCC) may increase our understanding of the complex biology of this disease.

**Methods:**

Tumor samples were obtained from untreated HNSCC patients undergoing surgery. Patients were classified according to pathologic lymph node status (positive or negative) or tumor recurrence (recurrent or non-recurrent tumor) after treatment (surgery with neck dissection followed by radiotherapy). Using microarray gene expression, we screened tumor samples according to modules comprised by genes in the same pathway or functional category.

**Results:**

The most frequent alterations were the repression of modules in negative lymph node (N0) and in non-recurrent tumors rather than induction of modules in N+ or in recurrent tumors. N0 tumors showed repression of modules that contain cell survival genes and in non-recurrent tumors cell-cell signaling and extracellular region modules were repressed.

**Conclusions:**

The repression of modules that contain cell survival genes in N0 tumors reinforces the important role that apoptosis plays in the regulation of metastasis. In addition, because tumor samples used here were not microdissected, tumor gene expression data are represented together with the stroma, which may reveal signaling between the microenvironment and tumor cells. For instance, in non-recurrent tumors, extracellular region module was repressed, indicating that the stroma and tumor cells may have fewer interactions, which disable metastasis development. Finally, the genes highlighted in our analysis can be implicated in more than one pathway or characteristic, suggesting that therapeutic approaches to prevent tumor progression should target more than one gene or pathway, specially apoptosis and interactions between tumor cells and the stroma.

## Background

Head and neck squamous cell carcinomas (HNSCC) are the seventh most common solid malignancy in the United States, accounting for more than 47,000 new cancer cases per year [1]. Surgery of patients clinically diagnosed with lymph node metastasis (N+) usually involves neck dissection, which can cause disfigurement, functional impairment, and pain. However, after histological examination, more than 30% of clinically N+ patients turn out to be pathologically metastasis-free (pN0) [2].

Clinically N0 patients may have occult node metastasis in up to 50% of the cases; in other words, these patients are pathologically N+ (pN+) [3]. Within two years of follow-up, these patients may develop metastatic disease [4]. On the other hand, around 50% of N0 patients do not have occult node metastasis. Therefore, many pN0 patients undergo neck dissection unnecessarily. Due to limitations in detecting lymph node metastasis before surgery, both pN+ and pN0 patients may receive inappropriate treatment. This indicates that a better understanding of the biology of HNSCC is urgently needed.

HNSCC also shows a great diversity in its clinical behavior after treatment, which cannot be predicted using the currently available clinical or pathological markers. Despite tumor complexity, many studies have unsuccessfully tested the prognostic value of single genes as markers for HNSCC. The most studied genes related to the onset of HNSCC are *epidermal growth factor receptor *(*EGFR*), *Cyclin D1*, *p53*, and Human Papilloma Virus (HPV), while the expression of matrix metallopeptidases (MMP) and vascular endothelial growth factor (VEGF) are associated with the metastatic phenotype (for review see references [5,6]). Despite the development of target therapies against EGFR and VEGFR, metastasis affects 40 to 50% of advanced HNSCC patients [7-9].

Microarray studies provide simultaneously measurement of thousands of genes, having therefore been used to identify many differentially expressed genes and expression profiles in HNSCC. For instance, a molecular signature of 102 genes was identified and independently validated for detection of lymph node metastasis with overall accuracy of 86% [10]. Later, numerous combinations of genes were used for predicting lymph node status, suggesting that molecular signatures based on a large number of genes are less likely to be biased towards specific samples [11]. Unfortunately, the use of expression profiles has not been incorporated into the clinic due to a lack of standards in experimental design and heterogeneous results [12].

According to the functional analysis of microarray data described by Segal et al. [13], a group of tumor samples can be characterized according to the behavior of modules. These modules include clusters of genes that belong to the same pathway or functional category [14], [15].

Cancer is a multifaceted phenomenon that involves activation and/or disruption of various cellular processes. Thus, the identification of pathways or groups of genes such as those involved in the development of lymph node metastasis and recurrent disease may help us understand the complex biology of HNSCC.

Previously, our group successfully applied the functional analysis strategy to identify altered modules in pre-malignant and adenocarcinomas of the stomach and esophagus [16]. Here we describe the application of this analysis to screen modules of genes with altered expression in poor prognosis HNSCC (positive lymph node or recurrent tumors). Our findings suggest that progression of HNSCC may be inhibited by loss of expression of genes related to cell survival and interactions with the stroma.

## Methods

### Patients and tissue samples

The study was approved by our institutional review board and all patients signed a pre-informed consent. Samples were obtained from surgery of patients with untreated HNSCC at the Head and Neck Surgery and Otorhinolaryngology Department of the Hospital AC Camargo (São Paulo, Brazil) (Table 1). During surgery, all patients were submitted to neck dissection. No patients received chemotherapy before or after surgery. All patients whose samples were used in the comparison between recurrent *versus *non-recurrent tumors were treated with adjuvant radiotherapy. Tumor samples were snap-frozen in liquid nitrogen. Before RNA extraction, diagnosis was confirmed by hematoxylin-eosin staining. Frozen samples were hand dissected for removal of normal cells, necrosis, and infiltrating inflammatory cells.

**Table 1:** Clinical information of patients with head and neck squamous cell carcinoma.

	pN+	pN0	Recurrent*	Non-recurrent*
Samples	61	20	20	27

Oral cavity (%, n)	55.7 (34)	65.0 (13)	50.0 (10)	44.4 (12)

Oropharynx (%, n)	44.3 (27)	35.0 (7)	35.0 (7)	40.7 (11)

Hypopharynx (%, n)	0	0	5.0 (1)	7.4 (2)

Larynx (%, n)	0	0	10.0 (2)	7.4 (2)

Age (mean in years**)	56.9 (23-85)	61.2 (35-88)	57.2 (39-74)	55.5 (23-78)

Male gender (%, n)	82.0 (50)	80.0 (16)	90.0 (18)	74.1 (20)

Smoking (%, n)	83.6 (51)	70.0 (14)	95.0 (19)	74.1 (20)

Alcohol (%, n)	67.2 (41)	50.0 (10)	80.0 (16)	63.0 (17)

pT3-4 (%, n)	73.8 (45)	50.0 (10)	80.0 (16)	59.3 (16)

Follow-up (years**)	0 - 9.03	0,01 - 7,80	0.37 - 3.47	2.41 - 9.03

Locoregional recurrence or	44.3 (27)	25.0 (5)		

distant metastasis (%, n)				

Note: *All patients were submitted to surgery and neck dissection, presented pathologic positive lymph node status, and were treated with adjuvant radiotherapy. Recurrence was considered as locoregional and/or distant metastasis after treatment (biopsy proven or by imaging). **minimum - maximum

### Microarray expression analysis

The experimental procedures and raw data are available at the Gene Expression Omnibus, according to the Minimum Information About a Microarray Experiment standards [GEO:GSE22243] [17]. Total RNA was extracted using Trizol (Invitrogen, Carlsbad, CA). RNA quality was assessed by spectrophotometry and gel electrophoresis. To be considered as high-quality, the RNA had to have a 260/280 ratio higher than 1.7 and an 18S/28S rRNA ratio ~2. Amplification of the mRNA was done using a T7-based protocol [18] and cDNA was indirectly labeled with Alexa Dye 555 or 647 (Invitrogen, Carlsbad, CA). Samples and a common RNA reference were hybridized overnight at 42°C in dye-swap to a 4,800-element in-house printed microarray [GEO:GPL8173] [19], enriched with cancer-related ESTs derived from the Human Cancer Genome Project [20], [21]. After washing, slides were scanned on a confocal laser scanner (ScanArray Express, Perkin-Elmer Life Sciences, Boston, MA) and data were extracted with ScanArray Express software.

### Functional modules analysis

The analysis was carried in R [22], [23] plus Bioconductor's maigesPack [24]. Background-subtracted spot intensities were normalized by Loess using span 0.4 and degree 2. Next, we searched for gene expression patterns associated with clinical/pathological parameters but which were not necessarily associated with individual samples, following the method described by Segal et al. [13]. For that, we defined functional modules according to the following databases: Biocarta [25], GeneDecks [26], Gene Ontology [27], and Kyoto Encyclopedia of Genes and Genomes Pathway Database (KEGGPD) [28]. 334 modules could be represented by the EST sequences spotted on the microarray chip (Additional File 1).

**Figure 1:**
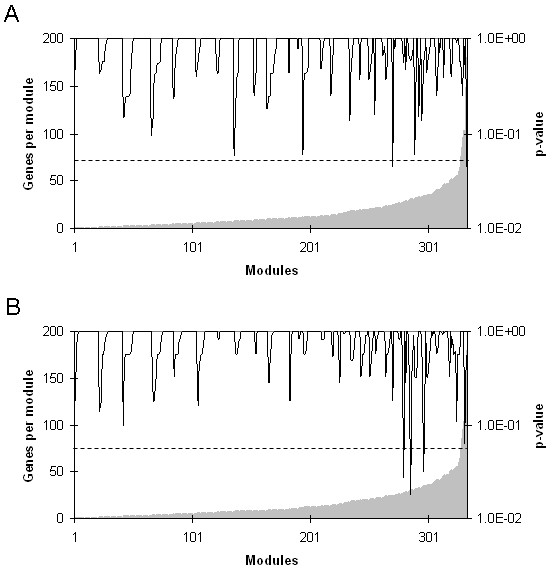
**Number of genes represented in each functional module and p- values for the repressed modules hypothesis**. HNSCC patients were classified according to pathologic lymph node status (positive or negative) or tumor recurrence (recurrent or non-recurrent tumor) after treatment (surgery with neck dissection followed by radiotherapy). Gene expression was assessed by microarray and functional module analysis was performed. Functional modules were defined according to the following databases: Biocarta], GeneDecks, Gene Ontology, and to the Kyoto Encyclopedia of Genes and Genomes Pathway Database (KEGGPD). A module is considered to be induced or repressed when it contains a fraction of induced or repressed genes that is higher than expected. The p-value of this fraction was calculated using hypergeometric distribution and a false discovery rate of 5% was applied to correct multiple testing. A - Negative lymph node (pN0). B - Non-recurrent tumors. These figures are representative of those for positive lymph node (pN+) and for recurrent tumors, as well as for the induced modules hypothesis.

According to Segal and colleagues [13], a gene in each sample is induced when its expression is two times higher than the average expression in all samples, and is repressed when its expression is two times lower than the same average. Next, we identified the modules in each sample containing a higher than expected fraction of induced or repressed genes. For the corresponding hypothesis test, the fraction of induced and repressed genes within each module in a sample would, under the null hypothesis, show a hypergeometric distribution. The hypergeometric distribution is used to calculate a p-value for this fraction and a 5% FDR is used to correct for multiple testing. Likewise, a module is considered to be induced/repressed in a group of samples when a higher than expected frequency of samples shows induction/repression of the module. Although the size of the modules ranged from 1 to 180 genes, the p-value of altered modules was not related to the number of gene in each of them (Figure 1).

### Quantitative RT-PCR and statistical analysis

Expression of selected genes from altered modules was analyzed by quantitative reverse transcription-polymerase chain reaction (qRT-PCR) in a ABI Prism^® ^7300 Sequence Detection System (Applied Biosystems, Foster City, CA) using 1X SYBR Green PCR Master Mix (Applied Biosystems, Foster City, CA). Three constitutive genes (BCR2, HBMS, and HPRT1) were used to calculate a normalization factor through GeNorm [29]. Primer efficiency was used to calculate the relative expression to the RNA reference [30]. Median expression in the analyzed groups was compared using Mann-Whitney test. Logistic regression model was employed to identify independent risk factor for lymph node metastasis (pN+). Kaplan-Meier method was used to analyze recurrence-free survival and curves were compared with the log-rank test. Cox proportional hazards model was employed to identify independent risk factors for recurrence.

## Results

Two types of clinical parameters were used to assess the expression pattern of functional modules associated with poor prognosis in HNSCC: lymph node metastasis prior to surgery and recurrence after treatment.

### Functional modules related to lymph node metastasis

To analyze the pattern of functional expression associated with lymph node metastasis, samples from primary squamous cell carcinoma of the oral cavity and oropharynx were collected from previously untreated patients and classified according pathologic status as either negative (pN0) or positive (pN+) for lymph node metastasis.

Functional modules analysis was performed, which quantify the enrichment of group of genes in samples profiled by microarray. Specifically, it tests whether the fraction of over- or under-expressed genes in each sample is higher than the randomly expected according to hypergeometric distribution. Next, the analysis assesses whether particular group of samples (such as pN0 or pN+) preferentially over- or under-express a certain module of genes.

Among 334 tested modules, alterations with statistical significance were found in four modules: Kallikrein pathway, Protein binding, Regulation of apoptosis, and Metabolism of xenobiotics by cytochrome P450. As only a subset of genes in a module may contribute to its expression signature, core results are shown in Table 2 (complete list of altered modules and its genes can be found in Additional File 2).

**Table 2:** Genes found in altered modules of head and neck squamous cell carcinoma (HNSCC).

Module	Status	Gene symbol	p-value
**Negative lymph node (20 samples)**			

Protein biding	repressed	FOSB	4.1E-31
		
		KLK6	5.7E-18
		
		AREG	1.3E-11
		
		CCL20	4.0E-08
		
		IL1B	7.5E-05
		
		MYC	2.0E-04
		
		INHBA	7.0E-03
		
		EGFR	1.4E-02
		
		AKT1	2.1E-02

Regulation of apoptosis	repressed	SPHK1	9.0E-09
		
		INHBA	9.0E-09
		
		AKT1	9.7E-03

KLK pathway	induced	KLK13	4.0E-15
		
		KLK7	2.8E-05
		
		KLK6	1.5E-02
		
		SERPINA3	1.7E-02

**Positive lymph node (61 samples)**			

KLK pathway	repressed	KLK13	4.0E-15
		
		KLK7	2.8E-05
		
		KLK6	1.5E-02
		
		SERPINA3	1.7E-02

**Recurrent tumors (20 samples)**			

Cell-cell signaling	induced	IL1F9	4.1E-20
		
		AREG	5.5E-15
		
		INHBA	1.5E-12
		
		BST2	7.6E-10
		
		CCL20	2.1E-3
		
		KLK6	8.9E-3

**Non-recurrent tumor (27samples)**			

Cell-cell signaling	repressed	IL1F9	4.1E-20
		
		AREG	5.5E-15
		
		INHBA	1.5E-12
		
		BST2	7.6E-10
		
		CCL20	2.1E-3
		
		KLK6	8.9E-3

Extracellular region	repressed	INHBA	6.6E-15
		
		POSTN	3.0E-9
		
		KLK13	1.9E-6
		
		AREG	2.4E-6
		
		MMP13	7.3E-4
		
		KLK6	1.6E-2

HNSCC patients were classified according to pathologic lymph node status (positive or negative) or tumor recurrence (recurrent or non-recurrent tumor) after treatment (surgery with neck dissection followed by radiotherapy) and samples from primary tumors were collected. Gene expression was assessed by microarray and functional module analysis was performed. Functional modules were defined according to the following databases: Biocarta], GeneDecks, Gene Ontology, and to the Kyoto Encyclopedia of Genes and Genomes Pathway Database (KEGGPD).

The pN+ group showed repression in the Kallikrein pathway, whereas pN0 tumors showed induction of the same module. The pN0 group also under-express a panel of function-restricted gene related to Protein binding and Regulation of apoptosis (Table 2).

Next, expression of selected genes in altered modules was analyzed by qRT-PCR. Among the genes that most contributed to the repression of modules in the pN0 group, CCL20, EGFR, and INHBA were found to be down-regulated in this group compared to pN+ tumors (Table 3). Moreover, we found that INHBA up-regulation was associated with lymph node metastasis (N+) (odds ratio 5.6; 95% confidence interval 1.5 to 21.8; p-value = 0.012), as well as SERPINA3 down-regulation (odds ratio 4.0; 95% confidence interval 1.0 to 15.4; p-value = 0.044) (Table 4).

**Table 3:** Expression of genes found in altered modules of head and neck squamous cell carcinoma (HNSCC) assessed by quantitative reverse transcription-polymerase chain reaction (qRT-PCR).

Comparison	Gene	,p-value, Mann-Whitney test	Fold change
**Positive lymph node (61 samples) **	CCL20	0.009	3.40
**X**			
**Negative lymph node (20 samples)**	EGFR	0.006	1.70
	
	INHBA	0.014	2.05
	
	IL1B	0.07	1.38
	
	SERPINA3	0.07	-2.43
	
	AKT1	0.13	-
	
	MYC	0.17	-
	
	MCM2	0.22	-
	
	AREG	0.41	-
	
	PCNA	0.43	-
	
	KLK6	0.45	-
	
	FOSB	0.59	-

**Recurrent tumors (20 samples)**	BST2	0.014	-1.70
** X**			
**Non-recurrent tumors (27 samples)**	POSTN	0.058	2.47
	
	IL1F9	0.061	1.91
	
	CCL20	0.19	-
	
	INHBA	0.55	-
	
	AREG	0.67	-
	
	KLK6	0.76	-

HNSCC patients were classified according to pathologic lymph node status (positive or negative) or tumor recurrence (recurrent or non-recurrent tumor) after treatment (surgery with neck dissection followed by radiotherapy). Functional module analysis was performed on microarray gene expression data obtained from primary tumors. Expression of selected genes was assessed by qRT-PCR.

**Table 4:** Gene expression associated with lymph node metastasis and clinical outcome.

Logistic regression model				
**Response variable**	**Gene**	**Odds ratio**	C.I.* 95%	**p-value**
Lymph node metastasis**	INHBA (> 4.88)	5.6	1.5-21.8	0.012
	SERPINA3 (< 10.74)	4.0	1.0-15.4	0.044

**Cox proportional hazards model**				

**Response variable**	**Gene**	**Hazard ratio**	C.I.* 95%	**p-value**
Recurrence-free survival***	BST2 (< 3.07)	8.9	1.2-68.7	0.035
				

HNSCC patients were classified according to pathologic lymph node status (positive or negative) or tumor recurrence (recurrent or non-recurrent tumor) after treatment (surgery with neck dissection followed by radiotherapy) and samples from primary tumors were collected. Gene expression was assessed by quantitative reverse transcription-polymerase chain reaction (qRT-PCR). Logistic regression model was employed to identify independent risk factor for lymph node metastasis (pN+). Cox proportional hazards model was employed to identify independent risk factors for recurrence.

* C.I.: confidence interval

** Logistic regression model was adjusted by the following variables: CCL20 expression and primary site.

*** Cox proportional hazards model was adjusted by the following variables: CCL20 expression, primary site, and clinical staging.

### Functional modules related to locoregional and distant metastasis

In order to study the expression pattern of functional modules related to poor prognosis we also checked medical records for locoregional recurrence and/or distant metastasis. Follow-up was available for 47 pN+ patients. Twenty of them presented locoregional recurrence and/or distant metastases (biopsy proven or by imaging), and 27 were recurrence/metastasis-free after treatment.

Functional modules analysis showed alterations in the Cell-cell signaling and Extracellular region modules (Table 2, see Additional File 2 for complete list of altered modules). The cell-cell signaling module was repressed in non-recurrent tumors and induced in recurrent tumors. The Extracellular module was also repressed in non-recurrent tumors. As these two module share same genes, there is an overlap between the genes that most contributed to the repression of both modules.

Using qRT-PCR, we found that BST-2 was down-regulated in recurrent tumors (Table 3). Moreover, BST2 up-regulation was associated with recurrence-free survival (hazard ratio 8.9; 95% confidence interval 1.2 to 68.7; p-value = 0.035) (Table 4).

## Discussion

**Figure 2:**
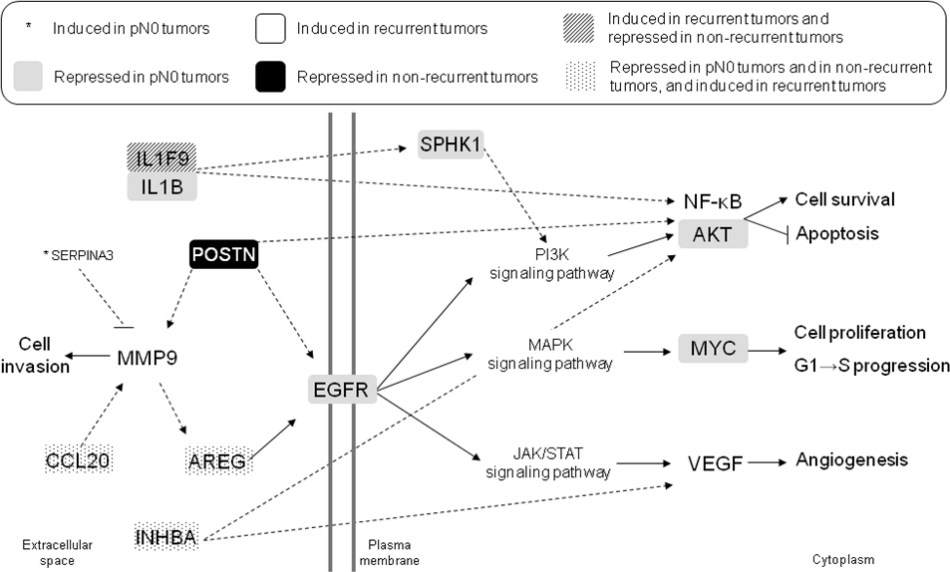
**Literature-based connections among genes in altered modules of head and neck squamous cell carcinoma (HNSCC)**. HNSCC patients were classified according to pathologic lymph node status (positive or negative) or tumor recurrence (recurrent or non-recurrent tumor) after treatment (surgery with neck dissection followed by radiotherapy). Gene expression was assessed by microarray and functional module analysis was performed. Functional modules were defined according to the following databases: Biocarta], GeneDecks, Gene Ontology, and to the Kyoto Encyclopedia of Genes and Genomes Pathway Database (KEGGPD). A module is considered to be induced or repressed when it contains a fraction of induced or repressed genes that is higher than expected. The p-value of this fraction was calculated using hypergeometric distribution and a false discovery rate of 5% was applied to correct multiple testing. Corrected p-values ranged between 4.1 × 10^-20 ^and 2.1 × 10^-2^. Filled arrows represent gene connections described in KEGGPD and dotted arrows, connections described elsewhere (see Discussion for references). Pointed arrows represent activation of gene expression or protein activity, and blunt arrows, inhibition.

To understand the complex biology of HNSCC, functional analysis of microarray data was used to identify groups of genes involved in the development of lymph node metastasis and recurrence of the disease. We used the strategy proposed by Segal et al [13] because, unlike other functional strategies, this analysis is not restricted to the identification of enriched modules from a list of differentially expressed genes; the functional modules analysis is capable of identifying which modules are induced or and which are repressed in each group of samples. In addition, our findings show that the use of existing biological knowledge in the form of module of genes is valuable tool for understanding tumors with different prognosis. The functional modules strategy characterized each condition by a particular combination of modules, providing important insights into the gene expression of primary tumors of patients with lymph node metastasis and recurrent disease. For instance, the altered modules harbor genes that are frequently studied in cancer but also suggest a role for some genes that have not been previously implicated in HNSCC or metastasis in general. Based on the literature and on the pathways presented by KEGGPD, many connections between these genes could be highlighted (Figure 2). These connections are summarized below.

Samples from patients with negative lymph node showed induction of the Kallikrein pathway. *SERPINA3 *belongs to the Kallikrein pathway, which is induced in pN0 tumors. *SERPINA3 *encodes the protease inhibitor alpha-1-antichymotrypsin, which was previously reported to inhibit MMP9 activation [31].

The protein binding module is repressed in pN0 tumors and it is represented by several genes. Inhibin beta A (*INHBA*) subunits form a homodimer, activin A, which stimulates *VEGF *gene expression in carcinoma cells [32] and in the v-akt murine thymoma viral oncogene homolog (AKT) pathway [33]. *SPHK1 *is a kinase that may promote growth proliferative advantage and resistance to apoptosis through activation of the extracellular signal-related kinases 1/2 (ERK1/2) and phosphatidylinositol 3-kinase (PI3K)/AKT pathways [34]. *SPHK1 *can also be activated by IL-1β [35]. *IL1F9 *gene is in the same locus as *IL1B*, suggesting a common origin [36]. *IL1F9 *promotes a signaling cascade similar to IL-1, activating NF-κB [37]. Cell survival is also represented here by *AKT1*, which phosphorylates and inactivates the apoptotic machinery (for review see reference [38]).

Epithelial growth factor receptor (*EGFR*), which has shown to be overexpressed in HNSCC in several studies, plays a critical role in HNSCC growth, invasion, metastasis, and angiogenesis (for review see reference [5]). Amphiregulin, the protein encoded by *AREG*, is a ligand of EGFR. In squamous cell carcinoma cells, amphiregulin is released by MMP cleavage. Moreover, *AREG *gene silencing or binding inhibition prevents EGFR phosphorylation and downstream events, such as cell proliferation, migration, and activation of the survival mediator AKT [39]. *CCL20*, the macrophage inflammatory protein-3α, mediates ERK1/2 activation through a MMP-dependent release of amphiregulin and transactivation of the EGFR. Again, blockade of amphiregulin reduced CCL20-mediated cell proliferation [40].

Considering these findings, we suggest that the tumor biology of pN0 is associated with repression of modules and genes related to proliferation and cell survival, which may be an important factor in lymph node involvement as apoptosis is one of the processes regulating metastasis (for review see reference [41]).

Gene expression profile of metastatic HNSCC has been recently described, and the most significantly altered transcripts in metastatic HNSCC were found to be associated with adhesion, mobility, and cell survival [42]. In our study non-recurrent tumors showed significantly repressed modules, namely cell-cell signaling and extracellular region, which contains *POSTN*. *POSTN *encodes periostin, which was implicated in several cellular functions in cancer. Periostin expression *in vitro *leads to EGFR and MMP9 expression [43]. Periostin may play a role in focal adhesion [44], and promote Akt activation, leading to tumor and endothelial cell survival [45]. Periostin may also be important in the epithelial-mesenchymal transition (EMT). *POSTN *expression led to EMT phenotype along with enhanced cell migration, invasion, and adhesion [43]. In HNSCC cell lines, periostin promoted distant and lymph node metastases in a murine model [46]. Periostin is also related to angiogenesis. In oral SCC, periostin+ samples had higher blood vessel density and recombinant periostin enhanced blood vessel formation *in vitro *[47]. Finally, it was shown that periostin is expressed by stromal cells [48] and by fibroblasts in cancer [49]. Therefore, it is possible that in non-recurrent tumors, in which the *POSTN *module is repressed, the stroma has fewer interactions with tumor cells, somehow disabling metastasis development.

One limitation of this study was that the connections among genes based on information from the literature were collected through different types of experiments done in several forms of cancer. Therefore, we cannot be sure that these connections really occur in HNSCC. These findings need to be validated by functional assays to assess the role they might have in complex and heterogeneous cancer cells. Moreover, the modules identified as induced or repressed were groups of genes that belong to the same functional category which do not necessarily represent pathways. However, according to Segal et al [13], many phenomena are sufficiently robust to be detected using functional modules analysis. So, the repression of functional categories may suggest that the main differences between two types of tumors (e. g. N0 and N+) are not in pathways *per se*, but in cellular functions instead.

In this study, we focused on the biology of the altered modules in pN0 vs. pN+ and in recurrent vs. non-recurrent tumors. The great advantage of the functional modules strategy is the increased comparability of results from different microarray studies [50]. Indeed, we identified altered modules with functions similar to those described by other authors using different approaches of functional gene expression in HNSCC. These functions include cell adhesion, cell death, cell growth and degradation of the extracellular matrix [10]. Up-regulation of genes that encode for MMP and for proteins related to inflammatory response was also found [12]. A supervised pathway-based analysis was performed in pN0 and pN+ HNSCC, indicating gene sets involved in extracellular matrix remodeling, hypoxia, and angiogenesis [50].

Three interesting observations can also be extrapolated from our dataset. First, the most frequent alterations found were the repression of modules in pN0 and in non-recurrent tumors. This also suggests that loss of various cellular functions may have a negative effect on the acquisition of the metastatic phenotype. Secondly, the tumor samples used here are not microdissected, so the data represent the gene expression of the tumor together with the stroma. Thus, the genes responsible for induction/repression of modules may show signaling between the microenvironment and the tumor cell. The understanding of how these interactions lead to tumor progression is critical for improving cancer treatment. Finally, many of the genes can be implicated in more than one pathway or more than one characteristic. Therefore, therapeutic approaches to prevent tumor progression should target more than one gene or pathway.

Functional modules analysis also provided interesting putative biomarkers that have not been previously implicated in HNSCC lymph node metastasis or recurrence. For instance, BST2 up-regulation was significantly associated with disease-free survival. BST2, also known as bone marrow stromal cell antigen 2, was implicated in many cellular functions in different cell types, such as development of B cell precursors [51], actin organization at the apical membrane of epithelial cells [52], and tethering HIV virions to cells [53]. Although BST2 function in HNSCC is unknown, BST2 expression may be used as prognostic marker to guide treatment. In addition, we found that INHBA up-regulation and SERPINA3 down-regulation are significantly associated with lymph node metastasis. This information would be very useful in routine care of HNSCC because the presence of lymph node metastasis prior to surgery is not easily determined by the currently avaible methods.

## Conclusions

The alterations identified here offer a comprehensive initial view of the molecular mechanisms of HNSCC, suggesting that loss of cell survival and interactions with the stroma inhibits HNSCC progression. Functional and/or *in vivo *studies may determine which genes, pathways, and gene set combinations have the greatest impact on cancer progression, which may contribute for the improvement of HNSCC treatment.

## Competing interests

The authors declare that they have no competing interests.

## Authors' contributions

AELC participated in the collection of clinical information, carried out the microarray experiments, participated in the data analysis and drafted the manuscript. ACQS organized and performed data analysis, and reviewed the manuscript. ALC participated in the collection of samples and clinical information, and helped on the interpretation of results. CMM helped in the experimental assays. LF participated in data analysis. LPK participated in the collection of samples and clinical information, and reviewed the manuscript. FAS helped in the collection of samples. EJN coordinated data analysis. LFLR conceived of the study and helped in the interpretation of results. AFC participated in the design and in the coordination of the study. All authors read and approved the final manuscript.

## Authors' information

Current Addresses:

AELC: Hospital Israelita Albert Einstein, Avenida Albert Einstein, 627, São Paulo, SP, 05652-900, Brazil

ALC: Hospital do Câncer de Barretos, Departamento de Cirurgia de Cabeça e Pescoço, Avenida Antenor Duarte Villela, 1331, Barretos, SP, 14784-400, Brazil

LFLR: Hospital Sírio-Libanês, Rua Adma Jafet, 91, São Paulo, SP, 01308-050, Brazil

## Pre-publication history

The pre-publication history for this paper can be accessed here:

http://www.biomedcentral.com/1755-8794/4/33/prepub

## Supplementary Material

Additional File 1 **Genes and modules present in microarray platform GPL8173**. list of all modules represented in the microarray platform and the complete list of genes in each represented module. Click here for file

Additional File 2 **Complete list of altered modules found in altered modules of head and neck squamous cell carcinoma (HNSCC)**. list of all altered modules and all genes representing them in HNSCC classified according to pathologic lymph node status (positive or negative) or tumor recurrence (recurrent or non-recurrent tumor). Click here for file
