# Supplementary material for: Functional microarray analysis suggests repressed cell-cell signaling and cell survival-related modules inhibit progression of head and neck squamous cell carcinoma
Source: BMC Med Genomics. 2011 Apr 13;4:33. doi: 10.1186/1755-8794-4-33 (PMC3095999; doi:10.1186/1755-8794-4-33)
Supplement: Additional File 2 — Complete list of altered modules found in altered modules of head and neck squamous cell carcinoma (HNSCC). list of all altered modules and all genes representing them in HNSCC classified according to pathologic lymph node status (positive or negative) or tumor recurrence (recurrent or non-recurrent tumor). [file 1755-8794-4-33-S2.DOC]

## Additional File 2. Complete list of genes found in altered modules in head and neck squamous cell carcinoma (HNSCC).

HNSCC patients were classified according to pathologic lymph node status (positive or negative) or tumor recurrence (recurrent or non-recurrent tumor) after treatment (surgery with neck dissection followed by radiotherapy). Gene expression was assessed by microarray and functional module analysis was performed. Functional modules were defined according to the following databases: Biocarta, GeneDecks, Gene Ontology, and Kyoto Encyclopedia of Genes and Genomes Pathway Database.

| Module | Status, score | Gene | p-value |
| --- | --- | --- | --- |
| KLK pathway | Repressed in pN+, -0.40 | KLK13 | 4.1E-15 |
|  | Induced in pN0, 0.40 | KLK7 | 2.9E-05 |
|  |  | KLK6 | 1.6E-02 |
|  |  | SERPINA3 | 1.8E-02 |
|  |  | EVPL | 4.3E-01 |
|  |  | SLPI | 8.6E-01 |
|  |  | SERPINB10 | 1.0E+00 |
|  |  | SCEL | 1.0E+00 |
|  |  | SERPINI1 | 1.0E+00 |
|  |  | SERPINB6 | 1.0E+00 |
| Protein Binding | Repressed in pN0, -0.15 | Gene | p-value |
|  |  | FOSB | 4.1E-31 |
|  |  | KLK6 | 5.8E-18 |
|  |  | AREG | 1.4E-11 |
|  |  | CCL20 | 4.0E-08 |
|  |  | EFNB2 | 9.2E-08 |
|  |  | MYH10 | 3.4E-05 |
|  |  | IL1B | 7.5E-05 |
|  |  | GPR56 | 1.0E-04 |
|  |  | NTRK2 | 2.0E-04 |
|  |  | MYC | 2.0E-04 |
|  |  | MCM2 | 2.0E-04 |
|  |  | CCL18 | 5.6E-03 |
|  |  | INHBA | 7.0E-03 |
|  |  | FXYD5 | 1.2E-02 |
|  |  | GRB7 | 1.4E-02 |
|  |  | EGFR | 1.4E-02 |
|  |  | RFX5 | 1.5E-02 |
|  |  | PLP2 | 2.1E-02 |
|  |  | AKT1 | 2.1E-02 |
|  |  | RGS16 | 1.7E-01 |
|  |  | PQBP1 | 1.7E-01 |
|  |  | E2F1 | 1.9E-01 |
|  |  | CUL4A | 1.9E-01 |
|  |  | MTSS1 | 1.9E-01 |
|  |  | IPO7 | 1.9E-01 |
|  |  | EPHB2 | 1.9E-01 |
|  |  | EDN3 | 1.9E-01 |
|  |  | NCOA6 | 1.9E-01 |
|  |  | FLOT2 | 2.1E-01 |
|  |  | NCOR1 | 2.1E-01 |
|  |  | IL6 | 2.1E-01 |
|  |  | NCOR2 | 2.3E-01 |
|  |  | PAWR | 2.3E-01 |
|  |  | POP7 | 2.3E-01 |
|  |  | MCM7 | 2.3E-01 |
|  |  | PRKDC | 2.3E-01 |
|  |  | MEF2A | 2.5E-01 |
|  |  | COTL1 | 2.5E-01 |
|  |  | FBLN5 | 2.5E-01 |
|  |  | AES | 3.0E-01 |
|  |  | MCM5 | 3.0E-01 |
|  |  | MBD2 | 3.0E-01 |
|  |  | ELF4 | 3.0E-01 |
|  |  | GANAB | 7.6E-01 |
|  |  | NCOA4 | 7.6E-01 |
|  |  | CECR1 | 7.6E-01 |
|  |  | GNA13 | 7.6E-01 |
|  |  | BRAP | 7.6E-01 |
|  |  | RBL2 | 7.6E-01 |
|  |  | HCK | 7.6E-01 |
|  |  | RAB5B | 7.6E-01 |
|  |  | KLHL5 | 7.6E-01 |
|  |  | HAT1 | 7.6E-01 |
|  |  | CUL3 | 7.6E-01 |
|  |  | BIRC2 | 7.6E-01 |
|  |  | GGA1 | 7.6E-01 |
|  |  | EPS8 | 7.6E-01 |
|  |  | FLOT1 | 7.6E-01 |
|  |  | MDN1 | 7.6E-01 |
|  |  | RGS2 | 7.6E-01 |
|  |  | NXF1 | 7.6E-01 |
|  |  | CAPZB | 7.6E-01 |
|  |  | LRP6 | 7.6E-01 |
|  |  | CD86 | 7.6E-01 |
|  |  | CCL25 | 7.6E-01 |
|  |  | ECM2 | 7.6E-01 |
|  |  | NAGK | 7.6E-01 |
|  |  | FEZ2 | 7.6E-01 |
|  |  | GFRA1 | 7.6E-01 |
|  |  | DOCK1 | 7.6E-01 |
|  |  | PENK | 7.6E-01 |
|  |  | BCL9 | 7.6E-01 |
|  |  | BAT5 | 7.6E-01 |
|  |  | PHF12 | 7.6E-01 |
|  |  | DEK | 7.6E-01 |
|  |  | GDF15 | 7.6E-01 |
|  |  | E2F2 | 7.6E-01 |
|  |  | ABI3 | 7.6E-01 |
|  |  | GGA3 | 7.6E-01 |
|  |  | KLF7 | 7.6E-01 |
|  |  | NUDT5 | 7.6E-01 |
|  |  | IRAK4 | 7.6E-01 |
|  |  | MCRS1 | 7.6E-01 |
|  |  | ILF3 | 7.6E-01 |
|  |  | APC | 7.6E-01 |
|  |  | LSM5 | 7.6E-01 |
|  |  | KISS1 | 7.6E-01 |
|  |  | PAK1 | 7.6E-01 |
|  |  | GRK5 | 7.6E-01 |
|  |  | IRF3 | 7.6E-01 |
|  |  | FAF1 | 7.6E-01 |
|  |  | JAK2 | 7.6E-01 |
|  |  | DTNA | 7.6E-01 |
|  |  | NUP98 | 7.6E-01 |
|  |  | LIMK1 | 7.6E-01 |
|  |  | MARK2 | 7.6E-01 |
|  |  | PARD3 | 7.6E-01 |
|  |  | CRK | 7.6E-01 |
|  |  | CRSP6 | 7.6E-01 |
|  |  | ERCC3 | 7.6E-01 |
|  |  | FGFR1 | 7.6E-01 |
|  |  | PRTN3 | 7.6E-01 |
|  |  | BCL10 | 7.6E-01 |
|  |  | LMAN1 | 7.6E-01 |
|  |  | EP300 | 7.6E-01 |
|  |  | ERG | 7.6E-01 |
|  |  | CCR1 | 7.6E-01 |
|  |  | KIF1B | 7.6E-01 |
|  |  | ATF2 | 7.6E-01 |
|  |  | FBXL2 | 7.6E-01 |
|  |  | BRD8 | 7.6E-01 |
|  |  | PEX12 | 7.6E-01 |
|  |  | AATF | 7.6E-01 |
|  |  | MATK | 7.6E-01 |
|  |  | APBB3 | 7.6E-01 |
|  |  | CD72 | 7.6E-01 |
|  |  | EVL | 7.6E-01 |
|  |  | MBD3 | 7.6E-01 |
|  |  | ARNT2 | 7.6E-01 |
|  |  | COG1 | 7.6E-01 |
|  |  | NTRK3 | 7.6E-01 |
|  |  | AHR | 7.6E-01 |
|  |  | CBL | 7.6E-01 |
|  |  | PEX10 | 7.6E-01 |
|  |  | BTBD1 | 7.6E-01 |
|  |  | PLDN | 7.6E-01 |
|  |  | CUL4B | 7.6E-01 |
|  |  | CTCF | 7.6E-01 |
|  |  | LRP1B | 7.6E-01 |
|  |  | CLIC1 | 7.6E-01 |
|  |  | MKKS | 7.6E-01 |
|  |  | MYT1 | 7.6E-01 |
|  |  | CTGF | 7.6E-01 |
|  |  | GRLF1 | 7.6E-01 |
|  |  | FHOD1 | 7.6E-01 |
|  |  | RFC2 | 7.6E-01 |
|  |  | E2F4 | 7.6E-01 |
|  |  | DEDD2 | 7.6E-01 |
|  |  | LIMD1 | 7.6E-01 |
|  |  | HDAC6 | 7.6E-01 |
|  |  | LIF | 7.6E-01 |
|  |  | PEX5 | 7.6E-01 |
|  |  | NPY1R | 7.6E-01 |
|  |  | RIPK1 | 7.6E-01 |
|  |  | LBR | 7.6E-01 |
|  |  | CDK6 | 7.6E-01 |
|  |  | DDEF1 | 7.6E-01 |
|  |  | NCOA1 | 7.6E-01 |
|  |  | NFKB2 | 7.6E-01 |
|  |  | CREB3 | 7.6E-01 |
|  |  | EPOR | 7.6E-01 |
|  |  | MTMR2 | 7.6E-01 |
|  |  | MKRN1 | 7.6E-01 |
|  |  | MBD1 | 7.6E-01 |
|  |  | RAB5A | 7.6E-01 |
|  |  | CD5 | 7.6E-01 |
|  |  | DHX9 | 7.6E-01 |
|  |  | ITSN2 | 7.6E-01 |
|  |  | DGKD | 7.6E-01 |
|  |  | CASP9 | 7.6E-01 |
|  |  | IRS1 | 7.6E-01 |
|  |  | PLCG2 | 7.6E-01 |
|  |  | ELMO1 | 7.6E-01 |
|  |  | GAB1 | 7.6E-01 |
|  |  | JAG1 | 7.6E-01 |
|  |  | ITSN1 | 7.6E-01 |
|  |  | ELK4 | 7.6E-01 |
|  |  | BID | 7.6E-01 |
|  |  | HAX1 | 7.6E-01 |
|  |  | DDX20 | 7.6E-01 |
|  |  | RBM9 | 7.6E-01 |
|  |  | NCK1 | 7.6E-01 |
|  |  | DAPK1 | 7.6E-01 |
|  |  | LCK | 7.6E-01 |
|  |  | CD59 | 7.6E-01 |
|  |  | PRLR | 7.6E-01 |
|  |  | HSF2 | 7.6E-01 |
|  |  | NCK2 | 7.6E-01 |
|  |  | MEN1 | 7.6E-01 |
|  |  | MARK4 | 7.6E-01 |
| Regulation of apoptosis | Repressed in pN0, -0.15 | SPHK1 | 9.0E-09 |
|  |  | INHBA | 9.0E-09 |
|  |  | AKT1 | 9.7E-03 |
|  |  | TIA1 | 1.7E-01 |
|  |  | STK4 | 7.6E-01 |
|  |  | CUL3 | 7.6E-01 |
|  |  | OPA1 | 7.6E-01 |
|  |  | TRAF3 | 7.6E-01 |
|  |  | FAF1 | 7.6E-01 |
|  |  | PAWR | 7.6E-01 |
|  |  | ERCC3 | 7.6E-01 |
|  |  | SSR3 | 7.6E-01 |
|  |  | BCL10 | 7.6E-01 |
|  |  | BIRC6 | 7.6E-01 |
|  |  | CUL4A | 7.6E-01 |
|  |  | TRADD | 7.6E-01 |
|  |  | DEDD2 | 7.6E-01 |
|  |  | CASP6 | 7.6E-01 |
|  |  | HIPK3 | 7.6E-01 |
|  |  | FAIM2 | 7.6E-01 |
|  |  | BID | 7.6E-01 |
|  |  | DAPK1 | 7.6E-01 |
|  |  | EI24 | 7.6E-01 |
|  |  | PRLR | 7.6E-01 |
|  |  | TGFB1 | 7.6E-01 |
| Metabolism of xenobiotics | Induced in pN0 tumors, 0.35 | AKR1C2 | 2.2E-13 |
| by cytochrome P450 |  | ADH5 | 9.7E-01 |
|  |  | EPHX1 | 9.8E-01 |
|  |  | CYP2S1 | 9.9E-01 |
|  |  |  |  |
| Cell-cell signaling | Induced in recurrent tumors, 0.50 | IL1F9 | 4.1E-20 |
|  | Repressed in non-recurrent | AREG | 5.5E-15 |
|  | Tumors, -0.44 | INHBA | 1.5E-12 |
|  |  | BST2 | 7.6E-10 |
|  |  | CCL20 | 2.1E-03 |
|  |  | KLK6 | 8.9E-03 |
|  |  | TGFA | 1.6E-01 |
|  |  | CCL18 | 1.9E-01 |
|  |  | GPR56 | 3.3E-01 |
|  |  | IL1B | 3.7E-01 |
|  |  | EDN3 | 4.4E-01 |
|  |  | WIF1 | 5.2E-01 |
|  |  | EFNB2 | 5.8E-01 |
|  |  | IL6 | 7.2E-01 |
|  |  | TGFB1 | 9.0E-01 |
|  |  | CD86 | 9.6E-01 |
|  |  | IL15 | 9.6E-01 |
|  |  | PENK | 9.6E-01 |
|  |  | NRP1 | 9.6E-01 |
|  |  | GDF15 | 9.6E-01 |
|  |  | MERTK | 9.6E-01 |
|  |  | DTNA | 9.6E-01 |
|  |  | SSR3 | 9.6E-01 |
|  |  | CCR1 | 9.6E-01 |
|  |  | KIF1B | 9.6E-01 |
|  |  | LARGE | 9.6E-01 |
|  |  | PLDN | 9.6E-01 |
|  |  | BSN | 9.6E-01 |
|  |  | CD97 | 9.6E-01 |
|  |  | GRIK5 | 9.6E-01 |
|  |  | LIF | 9.6E-01 |
|  |  | TGFB3 | 9.6E-01 |
|  |  | NPTX1 | 9.6E-01 |
|  |  | SCN1B | 9.6E-01 |
|  |  | TSHR | 9.6E-01 |
| Extracellular region | Repressed in non-recurrent | INHBA | 6.6E-15 |
|  | Tumors, -0.48 | POSTN | 3.0E-09 |
|  |  | KLK13 | 2.0E-06 |
|  |  | AREG | 2.4E-06 |
|  |  | MMP13 | 7.3E-04 |
|  |  | FSTL1 | 1.6E-02 |
|  |  | KLK6 | 1.7E-02 |
|  |  | CCL20 | 2.2E-01 |
|  |  | IL1B | 4.3E-01 |
|  |  | MMP9 | 5.2E-01 |
|  |  | LAMA4 | 6.2E-01 |
|  |  | FBLN2 | 7.4E-01 |
|  |  | IL6 | 8.1E-01 |
|  |  | NELL2 | 8.2E-01 |
|  |  | EGFR | 8.5E-01 |
|  |  | FBN1 | 8.5E-01 |
|  |  | CTGF | 8.5E-01 |
|  |  | CASK | 8.9E-01 |
|  |  | JAG1 | 8.9E-01 |
|  |  | ECM2 | 9.4E-01 |
|  |  | MMP2 | 9.5E-01 |
|  |  | IL15 | 9.6E-01 |
|  |  | FBLN5 | 9.6E-01 |
|  |  | KLK3 | 9.8E-01 |
|  |  | GDF15 | 9.8E-01 |
|  |  | LOXL2 | 9.8E-01 |
|  |  | IL16 | 9.8E-01 |
|  |  | MMP1 | 9.8E-01 |
|  |  | FVT1 | 9.8E-01 |
|  |  | MMP19 | 9.8E-01 |
|  |  | GPC1 | 9.8E-01 |
| TGF-β signaling pathway, | Repressed in non-recurrent | INHBA | 9.1E-11 |
|  | tumors, -0.22 | PPP2R2C | 3.0E-10 |
|  |  | THBS4 | 2.4E-05 |
|  |  | PPP2R1A | 1.5E-01 |
|  |  | MYC | 1.6E-01 |
|  |  | TGFB1 | 1.9E-01 |
|  |  | FST | 2.2E-01 |
|  |  | PPP2R2A | 4.3E-01 |
|  |  | SMURF1 | 5.6E-01 |
|  |  | RBL2 | 8.5E-01 |
|  |  | ACVR1 | 8.5E-01 |
|  |  | SMAD7 | 8.5E-01 |
|  |  | SMAD3 | 8.5E-01 |
|  |  | ACVR2A | 8.5E-01 |
|  |  | ID2 | 8.5E-01 |
|  |  | LTBP1 | 8.5E-01 |
|  |  | SMAD4 | 8.5E-01 |
|  |  | EP300 | 8.5E-01 |
|  |  | ACVR1C | 8.5E-01 |
|  |  | BMP8B | 8.5E-01 |
|  |  | ROCK2 | 8.5E-01 |
|  |  | E2F4 | 8.5E-01 |
|  |  | TGFB3 | 8.5E-01 |
|  |  | CREBBP | 8.5E-01 |
|  |  | THBS3 | 8.5E-01 |
|  |  | SP1 | 8.5E-01 |
|  |  | ACVR1B | 8.5E-01 |
|  |  | PPP2R1B | 8.5E-01 |
